# Meeting Patients with Hepatitis C Where They Are: Lessons from the HIV Epidemic in the USA

**DOI:** 10.1007/s11606-026-10629-7

**Published:** 2026-08-17

**Authors:** Anna C. Scialli, Tara Vijayan

**Affiliations:** 1 Division of General Internal Medicine, Department of Medicine, University of California, Los Angeles, CA USA; 2 Division of Infectious Diseases, Department of Medicine, University of California, Los Angeles, CA USA

**Keywords:** hepatitis C, HIV, antiretroviral therapy, health equity

## Abstract

Despite the advent of well-tolerated and curative direct-acting antivirals (DAAs) for hepatitis C virus (HCV), rates of acute HCV infections have increased in the past decade, persistently falling short of national eradication goals. HCV elimination strategies have adopted the care cascade, a framework originating in HIV care, to assess where patients are lost at each step of diagnosis, linkage to care, receipt of DAAs, and achievement of sustained virologic response. Interventions aimed at strengthening the HIV care cascade, including community-based testing, rapid initiation of treatment, and federal funding for treatment and housing, have been instrumental in increasing the number of people living with HIV who achieve viral suppression. Widescale uptake of HCV treatment, meanwhile, continues to face barriers, from payor restrictions to provider hesitance to treat patients who use drugs, despite evidence demonstrating high rates of treatment completion among this population. HIV care has critically reframed treatment nonadherence as a need for more support, rather than as a value judgment and as a reflection of the patient’s ability to receive treatment. We advocate for this approach to be applied to HCV care to provide broader and more equitable access to HCV treatment, and ultimately cure, particularly for marginalized populations such as people who use drugs (PWUD). We propose solutions for increasing HCV treatment uptake using lessons from HIV care, including using a test and treat approach to initiate DAAs during hospital admission.

*A 34-year-old male with a history of injection drug use and unstable housing was admitted to the hospital with subjective fever, generalized fatigue, and shortness of breath and was diagnosed with methicillin-sensitive Staphylococcus aureus endocarditis complicated by septic emboli. On screening, he was found to have hepatitis C virus (HCV) infection. He expressed a keen interest in starting treatment. He was admitted for 39 days for management of respiratory failure and tricuspid valve replacement and was transferred to a skilled nursing facility after discharge. During his hospitalization, he was started on glecaprevir-pibrentasvir, which he received from the institution’s specialty pharmacy, as it was not on the hospital formulary.*

Patients who use drugs (PWUD) and/or experience homelessness with chronic hepatitis C infection remain undertreated despite the availability of simplified treatment regimens.^1^ HCV elimination strategies have leveraged the care cascade framework used in the HIV epidemic, a framework that assesses treatment progress and completion through several progressive steps; for HCV, these steps include diagnosis, linkage to care, receipt of direct-acting antivirals (DAAs), and achievement of sustained virologic response.^1^ Despite these strategies, improvements in achieving these goals have fallen short when compared with the milestones achieved in HIV care.

Between 2015 and 2022, the USA saw an estimated twofold increase in acute HCV, yet only a third of insured individuals diagnosed with HCV receive treatment within a year of diagnosis.^2,3^ Greater strides have been made in HIV care, where the care cascade framework originated: 76% of those diagnosed with HIV receive any HIV care, and 65% are virally suppressed.^4^

At first glance, the markedly different natural histories of HIV and HCV suggest they have little in common. Notably, most patients with untreated HIV undergo progressive depletion of CD4 lymphocyte counts, leading to opportunistic infections and/or malignancies that define acquired immunodeficiency syndrome (AIDS) and are fatal within years.^5^ An estimated < 0.5% are considered “long-term non progressors” who can maintain a stable CD4 count without treatment for decades. Lifelong treatment with antiretroviral therapy has resulted in a dramatic increase in life expectancy. In contrast, patients with chronic HCV may remain relatively asymptomatic for decades.^6^ Chronic HCV infection gradually causes liver inflammation and fibrosis, with an estimated 5–10% incidence of cirrhosis after 20 years of infection and an estimated 1–3% rate of hepatocellular carcinoma after 30 years.^6,7^ At one point, HCV was the leading cause of liver transplant need in the USA, but the emergence of DAAs in the 2010s changed this epidemiology in select groups.^8^

Despite these differences, both viruses continue to affect the most vulnerable segments of our communities, those who have limited access to both prevention and treatment and those who have distinct socioeconomic disparities. It is not surprising then that the social, political, and public health histories of HIV and HCV overlap, revealing important lessons in forty years of progress in HIV elimination that can be applied toward the goal of HCV elimination.

## DISCOVERY AND IDENTIFICATION

First misidentified as a form of cancer, HIV was observed in New York as early as 1978.^9^ It was not until 1981 that a group of physicians in Los Angeles reported the first cases of Pneumocystis pneumonia among five men who have sex with men (MSM) in the Morbidity and Mortality Weekly Report; other medical journals soon followed.^10,11^ In 1984, the Centers for Disease Control and Prevention (CDC) first used the term AIDS to describe a disease “at least moderately predictive of a defect in cell-mediated immunity” caused by a retrovirus, labeled HTLV-III.^9,12,13^ In 1985, the Federal Drug Administration (FDA) approved the first blood test, an enzyme-linked immunosorbent assay, and blood banks began screening blood products.^14^ The discovery of HIV/AIDS was quickly politicized given its association with MSM; pejoratively referred to as a “gay cancer,” the diagnosis became profoundly stigmatizing, and was further used to marginalize sexual minorities.^9,15^ By 1990, over 100,000 people had died from AIDS in the USA, the majority of whom were MSM.^16^

HCV was first described in medical literature in 1975 among patients with transfusion-associated hepatitis that was not hepatitis A or B, prompting the name non-A, non-B hepatitis (NANBH), and was found to account for up to 90% of transfusion-associated hepatitis.^17,18^ While discovered in 1975, it was not until 1989 that HCV RNA was isolated and identified and an HCV antibody test was developed.^19^ By 1992, the blood supply was widely screened for HCV and it subsequently was estimated that there were approximately 230,000 HCV infections per year throughout the 1980s.^20^ The CDC initially encouraged screening among individuals with risk factors, including those who received blood transfusions prior to 1992 and PWUD.^21^ In 2012, following increasing evidence of HCV in the baby-boomer cohort (which accounted for over 75% of diagnosed infections between 1998 and 2008), the CDC recommended screening of all individuals born between 1945 and 1965.^20^ The advent of DAAs shifted the demographics over the next decade and by 2020 the CDC and US Preventive Services Task Force (USPSTF) recommended one-time screening for all adults given increasing rates among younger generations.^22^

## TREATMENT EVOLUTION

HIV treatment has profoundly evolved over four decades, from difficult to tolerate regimens requiring strict adherence to one-pill-a-day or injectable regimens offering viral suppression. While the first decade of discovery was marked by limited treatments, by 1996, David Ho and colleagues developed a combination regimen of protease inhibitors (PIs) and nonnucleoside reverse transcriptase inhibitor (NNRTIs), which combined with nucleoside reverse transcriptase inhibitors (NRTIs) became the foundation of modern HIV treatment and led to a dramatic decrease in morbidity and mortality.^23^ Initially referred to as highly active antiretroviral therapy (HAART), now simplified to ART, these early regimens were also marked by complicated dosing schedules, pill burden, significant side effects, and drug interactions. In the 2000s, single tablet combination regimens increased adherence and decreased toxicity and drug interactions, improving quality of life for people living with HIV.^24,25^ The most recent advancement in HIV treatment is long-acting injectables, such as cabotegravir and rilpivirine, which are well-tolerated and have shown high rates of viral suppression with reduced barriers to adherence.^26,27^

HCV treatment has similarly undergone a drastic evolution, from poorly tolerated drugs with low efficacy to now simple, curative medications. After the discovery of NANBH and prior to the identification of HCV, interferon-alpha was the mainstay of therapy, though it demonstrated low cure rates with high durations of treatment of up to 72 weeks.^18^ In 1991, the nucleoside analog ribavirin increased sustained virologic response compared to interferon-alpha alone. In 2001, more effective pegylated interferons with ribavirin became first-line treatment until 2011 but were poorly tolerated due to significant adverse effects such as flu-like symptoms, depression, and hemolytic anemia.^28^ These regimens required complicated dosing schedules, significant lab monitoring, and sometimes adjuvant therapies such as erythropoietin. The advent of DAAs in the 2010s revolutionized treatment, particularly the more recent pan-genotypic regimens first approved by the FDA in 2016.^18^ These medications, which include now first-line agents glecaprevir-pibrentasvir and sofosbuvir-velpatasvir, offer 8–12-week, once-daily dosing with rare side effects, no lab monitoring, and achieve cure in over 95% of those treated.

While current HCV treatment is well-tolerated and curative, many do not receive it. It is estimated up to 4 million people currently have HCV in the USA, yet fewer than one-third of insured adults receive treatment within a year of diagnosis.^3,29^ Given its natural history, we should expect many to advance to cirrhosis in the coming decades, a terminal diagnosis requiring liver transplantation. HCV now disproportionately impacts PWUD and individuals experiencing homelessness—populations frequently excluded from transplantation due to transplant boards’ requirements to demonstrate sobriety and social support. In the absence of accessible treatment, an HCV diagnosis therefore functionally constitutes a terminal illness.

**Figure 1:**
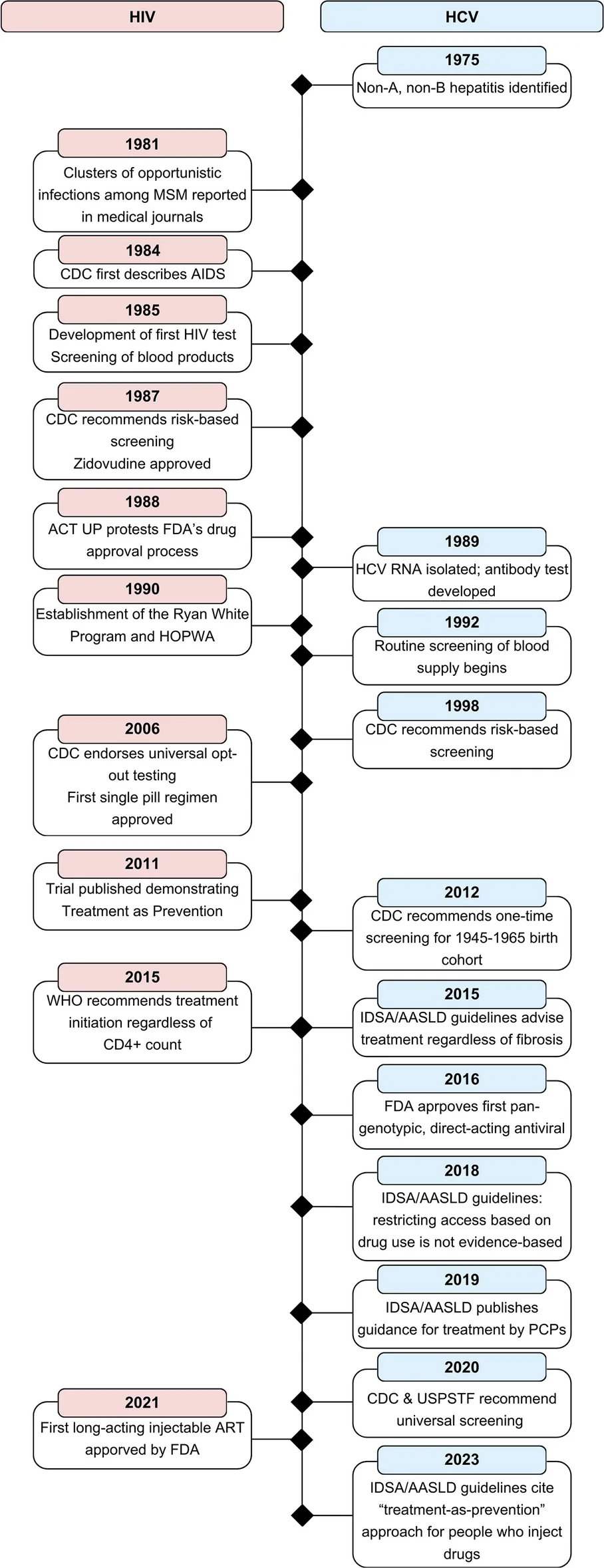
Key HIV and HCV testing and treatment advances, 1975–present.

**Figure 2:**
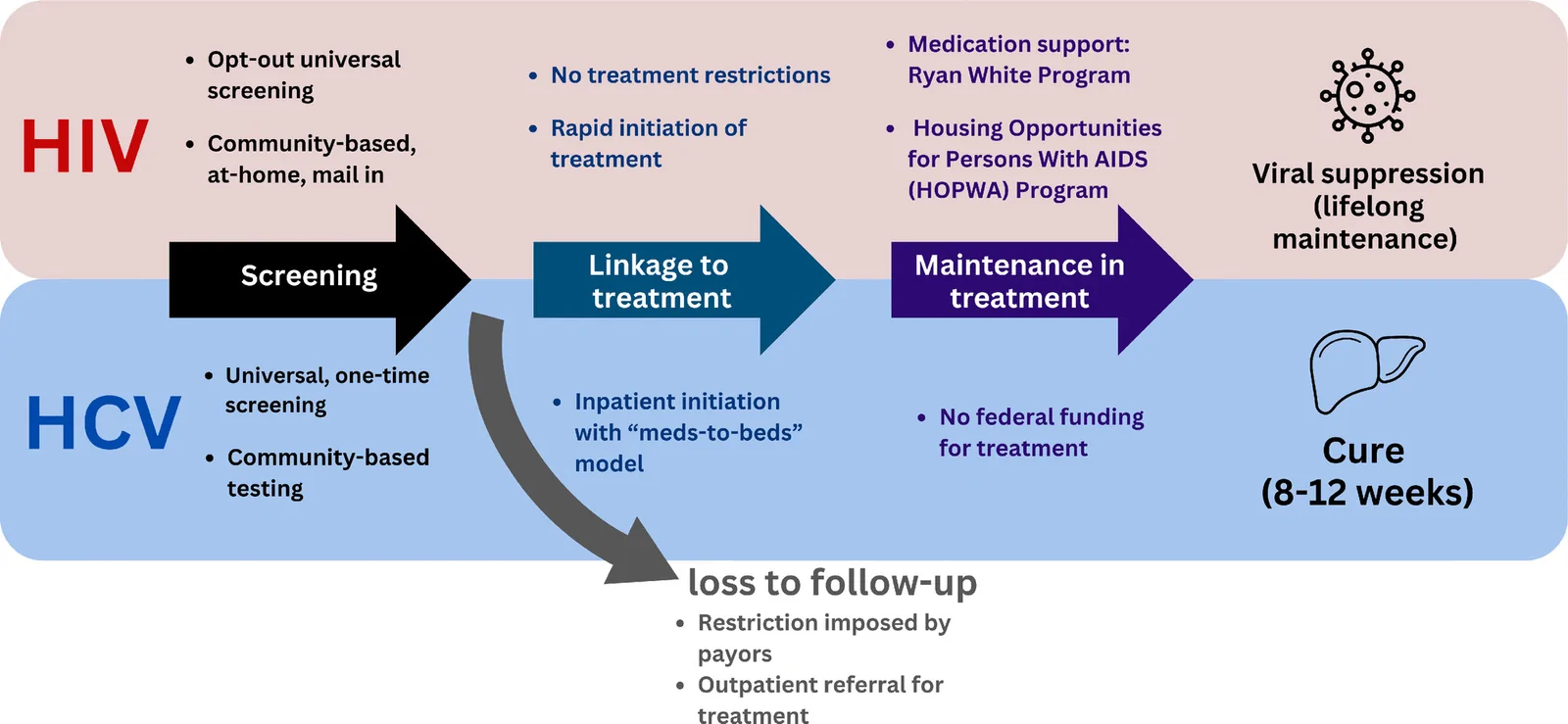
Differences in HIV and HCV treatment goals, policies, and strategies for maintenance in the care cascade.

In many ways, because treatment is short, well-tolerated, and curative, the barriers to HCV eradication should be far lower than what we expect for HIV. But there are several lessons to be learned from the history of HIV and the public health strategies that have been employed over the last four decades that could be used to move the needle forward, as evidenced by the timeline of HIV treatment advancements and their parallels with current HCV policy (Fig. 1). We outline these strategies, using the HIV care cascade as a framework (Fig. 2).

## TESTING AND TREATMENT STRATEGIES

Recreational drug use, and specifically injection drug use, is a primary driver of new HCV infections, especially among patients aged 18–40.^3^ PWUD face significant challenges in engaging in healthcare, including stigma and discrimination from healthcare professionals and socioeconomic barriers, highlighting a need for alternative strategies for the identification of HCV. At present, locations that treat substance use disorder (SUD) and sexually transmitted infections, including syringe service programs, have the highest proportion of HCV testing compared to other settings, such as emergency departments, ambulatory care, and prison or jail.^1^ While focusing on harm reduction programs remains an important strategy, this cannot be the only option to expand testing and treatment for HCV, particularly as syringe service programs lack federal support.

Initially, HIV testing was limited to blood donors and gradually expanded to include pregnant individuals, and those identified as having risk factors.^30^ In 2006, 21% of people with HIV were unaware of their status, and those who remained heavily stigmatized within their communities, including Black MSM, were disproportionately affected.^31^ As a result, the CDC recommended universal opt-out screening and launched several community-based educational efforts.^30^ By 2015, undiagnosed HIV infections in the USA decreased from 21 to 15%, with Black MSM more likely to report testing in the prior 12 months, a change attributed in part to the increased availability of low barrier testing outside of traditional health facilities.^32^

It is estimated that 55% of people with HCV are aware of their diagnosis, compared to an estimated 87% of people with HIV.^4,33^ The test and treat strategy for HIV, which not only confers benefit to individuals but reduces community viral loads and transmission substantially, has been implemented across various community-based and healthcare settings. These settings include brick and mortar clinics, mobile clinics, emergency rooms, community health fairs, and inpatient settings.^34^ We advocate for similar broad testing and treatment strategies for HCV, including initiating treatment in the inpatient setting, as was done for the above patient in the vignette. Because most hospitals are unwilling to purchase DAAs to keep in stock, we recommend a process that includes prescribing medications from a contracted specialty pharmacy that can purchase the drugs through 340B pricing and delivering it to the patient’s bedside by either storing it on the floor pyxis or keeping it in a secure location (similar to what might be done when a patient’s home medication is not available in the hospital pharmacy).^35^

## INSURANCE BARRIERS AND TREATMENT OPPORTUNITIES

Restrictions by insurance payors, including requiring periods of sobriety and restricting prescribing to specialty providers, persist in many states despite the recommendations of the Infectious Diseases Society of America (IDSA) and the American Association for the Study of Liver Disease (AASLD) to remove these barriers.^36,37^ States with Medicaid programs that impose sobriety and fibrosis restrictions are associated with fewer patients being treated with curative medication.^36^ Commercial insurance policies surrounding DAA coverage remain even more opaque, with private insurers denying over 50% of claims for HCV treatment.^38^

Until 2015, several international guidelines recommended ART initiation only for patients whose CD4+ counts fell below a threshold, despite studies demonstrating the benefit of early initiation of ART on mortality and a reduction in risk of transmission.^39^ Current national and international guidelines encourage the initiation of ART as soon as possible after diagnosis given this mortality benefit.^40^ National initiatives such as the Ryan White Comprehensive AIDS Resources Emergency (CARE) Act in 1990 have provided a federally funded safety net that covers ART for individuals living with HIV who are uninsured or under-insured, supporting 82% of uninsured individuals living with HIV and 38% with private insurance coverage who are unable to pay their premiums or co-pays.^41,42^ Interventions to overcome socioeconomic barriers and (re)engage people with HIV in care beyond the traditional healthcare setting of the outpatient clinic—such as during inpatient hospitalization—have increased treatment engagement and increased outpatient follow-up, especially among PWUD.^43,44^

Currently, no federally funded safety-net program exists to offer curative HCV treatment for uninsured individuals. While the policy landscape is becoming less restrictive, private insurers and state Medicaid agencies continue to restrict treatment despite clinical best practices. In the absence of a national funding stream to act as a safety net for individuals whose insurance coverage does not align with clinical best practices, such as Ryan White funding for HIV through the AIDS Drug Assistance Program (ADAP), rapid HCV initiation remains unavailable to many patients. Vulnerable patients with HCV, including those experiencing homelessness and those with SUD, have multiple hospital admissions, yet inpatient care remains siloed, only focusing on acute care needs. The Norway-based Opportunistic Treatment of Hepatitis C Infection Among Hospitalized People Who Inject Drugs (OPPORTUNI-C) Study, which compared inpatient initiation of HCV treatment among people who inject drugs to outpatient linkage to treatment, found that 68% of those who initiated treatment while hospitalized completed treatment compared to 35% of those who were referred to outpatient treatment.^45^ When given the right resources, including lowering the barrier imposed by third-party payors and/or accessing DAAs in the inpatient setting through processes described above, rapid initiation of HCV treatment at any point of engagement with the healthcare system can offer a unique opportunity to achieve cure.^46^

## PROVIDER ATTITUDE AND MOTIVATION TO TREAT

Provider bias regarding perceptions of non-adherence remains a barrier to initiating treatment for both HIV and HCV. A pertinent HIV study in 2017 study found that among providers who deferred ART initiation, adherence was a commonly cited factor; additional research has demonstrated that withholding treatment due to adherence concerns worsened racial and economic disparities in treatment initiation.^47,48^ Advocacy by communities disproportionately impacted by HIV has led to the reframing of HIV treatment nonadherence as a failure of systems in which marginalized groups face significant barriers.^41^ As a result, current CDC guidelines emphasize that adherence concerns due to homelessness, substance use, and mental health conditions are not justifiable reasons to defer ART in the context of the “simplicity, potency, and tolerability of contemporary ART.”^49^ Guidelines recommend leveraging person-centered social services to address social determinants of health that may negatively impact adherence. Federally funded programs designed to retain marginalized patients in care has reinforced this new approach to care. In addition to medication, case management services, and patient navigators through the Ryan White program, the US Department of Housing and Urban Development’s Housing Opportunities for Persons with AIDS (HOPWA) offers financial assistance for housing and has been shown to increase retention in care for people living with HIV.^50^

Despite the growing need for HCV treatment, the emergence of simple and well-tolerated DAAs, and IDSA/AASLD promotion of primary care prescribing of DAAs, rates of HCV treatment decreased from 2015 to 2019.^51,52^ Physicians often cite concerns about patient non-adherence as deterrents in offering HCV treatment, specifically for people who use drugs, even though studies have shown people who use drugs complete treatment over 96% of the time.^53,54^ These attitudes are apparent to patients, who describe stigma and discriminatory treatment from providers as barriers to initiation of and retention in care.^55^

While concerns over non-adherence persist for both HIV and HCV treatment, guidelines coupled with federally funded support services provide increased access to treatment for people living with HIV. Concerns about the risks of non-adherence have also decreased with the availability of antiretroviral regimens with a higher barrier to resistance. Similarly, newer DAAs for HCV are less vulnerable to emerging resistance and IDSA/AASLD guidelines explicitly cite that substance use is not a contraindication to HCV treatment.^37,56,57^ Unfortunately, this progress has not been matched by federal funding to support the socioeconomic conditions that impact HCV treatment adherence, as has been the case for HIV.

## CONCLUSION AND FUTURE DIRECTIONS

At present, inconsistent insurance payment policies and hesitancy to treat those most impacted by HCV continue to impede its elimination. We strongly advocate for expanded testing and treating strategies in HCV care to align with those that have been created for HIV care if we are to fully realize HCV elimination. These strategies include promoting treatment as a form of prevention regardless of the recency of HCV infection. Both testing and early treatment need to be expanded across diverse clinical and community-based settings, including the inpatient setting (Fig. 2).

Government funding plays an important role in eradication of HCV, as it historically has for HIV. Federally funded programs such as the Ryan White program and HOPWA have ensured access to life-saving HIV treatment regardless of socioeconomic status. The future of these programs remains uncertain as congressional budget cuts undermine critical safety net programs and public health initiatives. As of February 2026, 20 states were in the process of or considering tightening eligibility requirements of AIDS Drug Assistance Program (ADAP).^58^ The elimination of these programs would be devastating, with models estimating up to a 73% increase in new infections across 30 states if ADAP funding is eliminated.^59^ While no federally funded program for HCV treatment exists, an estimated 60% of people with HCV are covered by public insurance, such as Medicaid and Medicare, and the One Big Beautiful Bill in 2025 cut almost $1 trillion from Medicaid alone over the next decade.^36,60^

Lastly, grassroots political advocacy by members of communities most impacted by these illnesses has been central to the progress made in HIV treatment, leading to widespread adoption of what we now consider evidence-based care. The AIDS Coalition to Unleash Power (ACT UP) conducted revolutionary, public demonstrations at great risk to themselves given the stigmatization of HIV, protesting the high cost of zidovudine as well as the restriction of access by the FDA to experimental drugs to treat HIV.^15^ These efforts led to expanded access and accelerated drug-approval processes. Now, it is critical to center voices of those with HCV, including those with histories of substance use, to advocate for expanded access to HCV treatment.^61^ Peer support services, in which people with lived experience with hepatitis C treatment support those seeking treatment, have shown promising results.^62^ Drug user unions, collectives of people who use(d) drugs, advocate for policies that decrease stigma and increase access to nonjudgmental health services.^63^

As better tolerated, easier to take ART became increasingly available, and as it became apparent that treating people with HIV early conferred both individual and community level benefits, reducing the time to ART initiation became not simply a public health measure, but a moral imperative, with clinical guidelines strongly recommending same day initiation. Decades of public health research and advocacy in HIV have provided a roadmap for reducing barriers to care for HCV treatment, offering the opportunity for lifesaving, curative treatment within 12 weeks and tremendous public health benefit in decreasing community transmission. Achieving HCV elimination goals depends on the reframing of HCV through a public health and health equity lens in which we meet—and treat—patients where they are.
